# DR5‐Cbl‐b/c‐Cbl‐TRAF2 complex inhibits TRAIL‐induced apoptosis by promoting TRAF2‐mediated polyubiquitination of caspase‐8 in gastric cancer cells

**DOI:** 10.1002/1878-0261.12140

**Published:** 2017-10-27

**Authors:** Ling Xu, Ye Zhang, Xiujuan Qu, Xiaofang Che, Tianshu Guo, Ce Li, Rui Ma, Yibo Fan, Yanju Ma, Kezuo Hou, Danni Li, Xuejun Hu, Bofang Liu, Ruoxi Yu, Hongfei Yan, Jing Gong, Yunpeng Liu

**Affiliations:** ^1^ Department of Medical Oncology The First Hospital of China Medical University Shenyang China; ^2^ Key Laboratory of Anticancer Drugs and Biotherapy of Liaoning Province The First Hospital of China Medical University Shenyang China; ^3^ Department of Respiratory Medicine The First Hospital of China Medical University Shenyang China

**Keywords:** apoptosis, caspase‐8, Cbl‐b, c‐Cbl, TRAF2, TRAIL

## Abstract

Ubiquitination of caspase‐8 regulates TNF‐related apoptosis‐inducing ligand (TRAIL) sensitivity in cancer cells, and the preligand assembly complex plays a role in caspase‐8 polyubiquitination. However, whether such a complex exists in gastric cancer cells and its role in TRAIL‐triggered apoptosis is unclear. In this study, DR5, casitas B‐lineage lymphoma‐b (Cbl‐b)/c‐Cbl, and TRAF2 formed a complex in TRAIL‐resistant gastric cancer cells, and Cbl‐b and c‐Cbl were the critical adaptors linking DR5 and TRAF2. Treatment with TRAIL induced caspase‐8 translocation into the DR5‐Cbl‐b/c‐Cbl‐TRAF2 complex to interact with TRAF2, which then mediated the K48‐linked polyubiquitination of caspase‐8. The proteasome inhibitor bortezomib markedly enriched the p43/41 products of caspase‐8 activated by TRAIL, indicating proteasomal degradation of caspase‐8. Moreover, TRAF2 knockdown prevented the polyubiquitination of caspase‐8 and thus increased TRAIL sensitivity. In addition, the inhibition of Cbl‐b or c‐Cbl expression and overexpression of miR‐141 targeting Cbl‐b and c‐Cbl partially reversed TRAIL resistance by inhibiting the interaction between TRAF2 and caspase‐8 and the subsequent polyubiquitination of caspase‐8. These results indicate that the DR5‐Cbl‐b/c‐Cbl‐TRAF2 complex inhibited TRAIL‐induced apoptosis by promoting TRAF2‐mediated polyubiquitination of caspase‐8 in gastric cancer cells.

**Abbreviations**
Cbl‐b: casitas B‐lineage lymphoma‐b
CUL3: cullin 3
DISC: death‐inducing signaling complex
DR4: death receptor 4
DR5: death receptor 5
FADD: Fas‐associated protein with death domain
FLIP/L: long form of FLICE inhibitory protein
MT: mutant‐type
PARP: poly‐ADP‐ribose polymerase
PLAC: preligand assembly complex
RIP: receptor‐interacting protein
TNFAIP3: TNF‐α‐induced protein 3
TRAF2: tumor necrosis factor receptor‐associated factor 2
TRAIL: TNF‐related apoptosis‐inducing ligand
WT: wild‐type

## 1 Introduction

Many cancer therapies rely on triggering apoptotic pathways in tumor cells (Johnstone *et al*., 2008). Targeting TNF‐related apoptosis‐inducing ligand (TRAIL) receptors holds promise as one cancer therapeutic approach to selectively induce apoptosis in tumors (Herbst *et al*., 2010; Younes *et al*., 2010). Binding of TRAIL to death receptor 4 (DR4) or death receptor 5 (DR5) results in recruitment of Fas‐associated protein with death domain (FADD) and caspase‐8. This protein complex is known as the death‐inducing signaling complex (DISC). In the DISC, caspase‐8 is activated and cleaved to subsequently initiate apoptosis (Dickens *et al*., 2012). Our work and others have shown that gastric cancer cells are not sensitive to TRAIL (Fuentes *et al*., 2015; Xu *et al*., 2011, 2013a,b). However, the underlying mechanism has not been determined.

Recent studies showed that the ubiquitination of caspase‐8 regulates the sensitivity of cancer cells to TRAIL. The E3 ubiquitin ligase cullin 3 (CUL3)‐based polyubiquitination and p62‐dependent aggregation of caspase‐8 mediated extrinsic apoptosis signaling (Jin *et al*., 2009). In prostate cancer cells, TRAIL induced caspase‐8 polyubiquitination, and the inhibition of caspase‐8 polyubiquitination restored caspase‐8 activation and apoptotic signaling (Fiandalo *et al*., 2013). In addition, two forms of polyubiquitination have been described: K63 and K48‐linked polyubiquitination. K63‐linked polyubiquitination chains frequently control the function of proteins, while K48‐linked chains often promote proteasome‐mediated degradation of targeted proteins (Martin *et al*., 2014; Troppan *et al*., 2015). In colorectal cancer cells, tumor necrosis factor receptor‐associated factor 2 (TRAF2) directly mediates K48‐linked polyubiquitination on the large catalytic domain of caspase‐8 to induce the subsequent proteasomal degradation of caspase‐8, and thus relieves DR‐induced apoptosis (Gonzalvez *et al*., 2012). However, whether caspase‐8 is polyubiquitinated and the effect of polyubiquitinated caspase‐8 on TRAIL‐triggered apoptosis in gastric cancer cells have not been determined.

The DISC functions as the essential platform for TRAIL to activate caspase‐8 and subsequent apoptosis (De Miguel *et al*., 2015). However, another complex known as the preligand assembly complex (PLAC), containing TNF‐α‐induced protein 3 (TNFAIP3), receptor‐interacting protein (RIP), DR5, and TRAF2, has been found in glioblastoma. TRAIL results in the recruitment of caspase‐8 into the PLAC and inhibits caspase‐8 activation (Bellail *et al*., 2012). However, the existence of such complexes in gastric cancer cells and their potential role in TRAIL‐triggered apoptosis are unknown.

A recent study in hepatocellular cancer cells showed that TNFAIP3 could mediate the binding of polyubiquitinated RIP to caspase‐8 and inhibit caspase‐8 activation and TRAIL‐triggered apoptosis (Dong *et al*., 2012). Furthermore, our earlier work showed the involvement of the E3 ubiquitin ligase casitas B‐lineage lymphoma‐b (Cbl‐b) and its homologue c‐Cbl in TRAIL‐triggered gastric cancer cell apoptosis (Xu *et al*., 2009, 2012). Given that c‐Cbl is required for EGF‐induced K48‐linked polyubiquitination of ERBB4 cytoplasmic isoforms (Meijer *et al*., 2013), we therefore investigated whether Cbl‐b and c‐Cbl were components of a complex that regulates caspase‐8 polyubiquitination and hence influence the sensitivity of gastric cancer cells to TRAIL.

In this study, we demonstrated that DR5, Cbl‐b/c‐Cbl, and TRAF2 form a complex in TRAIL‐resistant gastric cancer cells and that Cbl‐b and c‐Cbl were the critical adaptors linking DR5 and TRAF2. TRAIL induced caspase‐8 translocation into DR5‐Cbl‐b/c‐Cbl‐TRAF2 complex to interact with TRAF2, and subsequently, TRAF2 mediated the polyubiquitination and degradation of caspase‐8. Moreover, the inhibition of TRAF2 or Cbl‐b/c‐Cbl expression and overexpression of miR‐141 targeting Cbl‐b/c‐Cbl partially reversed TRAIL resistance by inhibiting the interaction between TRAF2 and caspase‐8 and the subsequent polyubiquitination of caspase‐8.

## 2 Materials and methods

### 2.1 Cell cultures

The cells used were all from the Type Culture Collection of the Chinese Academy of Sciences (China). The cells were cultured in RPMI‐1640 medium containing 10% heat‐inactivated fetal bovine serum at 37 °C under an atmosphere of 95% air and 5% CO_2._

### 2.2 Antibodies and reagents

Anti‐FADD (mouse, monoclonal, sc‐271520, IB), anti‐DR5 (mouse, monoclonal, sc‐65314, IB), anti‐Cbl‐b (mouse, monoclonal, sc‐8006, IB), anti‐TRAF2 (mouse, monoclonal, sc‐137048, IB), anti‐c‐Cbl (mouse, monoclonal, sc‐1651, IB), anti‐Cbl‐b (rabbit, polyclonal, sc‐1705, IP), anti‐c‐Cbl (rabbit, polyclonal, sc‐170, IP), anti‐long form of FLICE inhibitory protein (FLIP/L; rabbit, polyclonal, sc‐8346, IB), and anti‐actin (rabbit, polyclonal, sc‐1616‐R, IB) antibodies were obtained from Santa Cruz Biotechnology (Santa Cruz, CA, USA). Anti‐caspase‐3 (rabbit, polyclonal, #9662, IB), anti‐caspase‐8 (mouse, monoclonal, #9746, IP and IB), anti‐caspase‐8 (rabbit, monoclonal, #4790, IB), K48 linkage‐specific polyubiquitin (rabbit, #8081, IB), K63 linkage‐specific polyubiquitin (rabbit, #5621, IB), anti‐caveolin‐1 (rabbit, monoclonal, #3267, IB), anti‐poly‐ADP‐ribose polymerase (PARP; rabbit, polyclonal, #9542, IB), anti‐RIP (rabbit, monoclonal, #3493, IB), anti‐CUL3 (rabbit, polyclonal, #2759, IB), anti‐DR5 (rabbit, monoclonal, #8074, IP and IB), and anti‐TRAF2 (rabbit, polyclonal, #4724, IP and IB) were purchased from Cell Signaling Technology (Danvers, MA, USA). Anti‐ubiquitin (linkage‐specific K48) antibody (Alexa Fluor 568; rabbit, monoclonal, ab208136, IF) was purchased from Abcam (Cambridge, UK). Anti‐caspase‐8 (mouse, monoclonal, 66093‐1‐lg, IF) was purchased from Proteintech Group Inc (Rosemont, IL, USA). Alexa Fluor 488 goat anti‐mouse IgG (H + L) was from Invitrogen (Waltham, MA, USA ). TRAIL was from Peprotech Asia (Rocky Hill, CT, USA).

### 2.3 Flow cytometry

The cells were collected and incubated with 5 μL Annexin V and 10 μL phosphatidylinositol for 15 min in the dark. Then, the samples were evaluated by flow cytometry, and the data were analyzed using cellquest software (Becton‐Dickinson, Franklin Lakes, NJ, USA).

### 2.4 Western blot and immunoprecipitation

Western blot and immunoprecipitation were performed as previously described (Xu *et al*., 2014). The cells were solubilized in 1% Triton lysis buffer. For immunoprecipitation (IP), cell lysates were mixed with the primary antibody and protein G/A–Sepharose beads at 4 °C overnight. The following primary antibodies were used in this study: DR5 (rabbit, #8074; Cell Signaling Technology), TRAF2 (rabbit, #4724; Cell Signaling Technology), Cbl‐b (rabbit, sc‐1705; Santa Cruz), or c‐Cbl (rabbit, sc‐170, Santa Cruz); protein A–Sepharose beads were added in these IP reactions. To IP caspase‐8 (mouse, #9746; Cell Signaling Technology) from cell lysates, protein G–Sepharose beads were added. The immunoprecipitated proteins were eluted by heat treatment at 100 °C for 5 min with 2× sampling buffer. Samples or protein lysates were separated by sodium dodecyl sulfate/polyacrylamide gel electrophoresis and electrophoretically transferred to a nitrocellulose membrane (Immobilon‐P; Millipore, Billerica, MA, USA). The membranes were blocked with 5% skim milk in TBST buffer at room temperature and incubated overnight at 4 °C with the indicated primary antibodies. After the appropriate secondary antibodies were added at room temperature, the proteins were detected with enhanced chemiluminescence reagent and visualized with the Electrophoresis Gel Imaging Analysis System (DNR Bio‐Imaging Systems, Neve Yamin, Israel).

### 2.5 Determination of caspase‐8 activity by absorption spectroscopy

Caspase‐8 activity was determined using a caspase colorimetric assay kit (KeyGen Biotech. Co. Ltd., NanJing, China). The cells were incubated with 100 ng·mL^−1^ TRAIL for 24 h. Then, the cells were suspended in 100 μL of chilled cell lysis buffer containing Hepes (50 mm, pH 7.4), Chaps (5 mm), PMSF (0.5 mm), and DTT (5 mm). After incubating on ice for 60 min, and centrifuging for 1 min in a microcentrifuge (20 000 ***g***), the sample (30 μL) was added to 2× reaction buffer (50 μL; 40 mm Hepes, pH 7.4, 3 mm Chaps, 10 mm DTT, and 4 mm EDTA), caspase‐8 substrate (10 μL, 4 mm), and ddH_2_O (10 μL) and then incubated at 37 °C for 1.5 h. The optical density (OD) was measured with a microplate reader (Model 550; Bio‐Rad Laboratories, Hercules, CA, USA).

### 2.6 Isolation of lipid rafts

Isolation of lipid rafts was performed as previously described (Xu *et al*., 2012). The gastric cancer cells were incubated with 100 ng·mL^−1^ TRAIL for 30 min. Then, the cells were solubilized in 150 μL of prechilled TXNE buffer (50 mm Tris/HCl pH 7.4, 150 mm NaCl, 5 mm EDTA, and 0.1% Triton X‐100) containing protease inhibitors (chymostatin, leupeptin, antipain, and pepstatin, at 25 mg·mL^−1^ each) for 30 min on ice. Subsequently, the cells were scraped off, extracted, and moved into 35% OptiPrep (Axis‐shield, Norway, Europe) in polyallomer ultratubes (Sorvall Instruments, Livonia, MI, USA) by adding 210 μL of 60% OptiPrep/0.1% Triton X‐100. Then, the cell lysates were covered with 3.5 mL 30% OptiPrep in TXNE buffer and 300 μL TXNE buffer. After spin (4 h, 20 0000 ***g***, 4 °C) in the ultracentrifuge (Sorvall/Kendro, Asheville, NC, USA), six fractions were collected from the top. The proteins in fractions 1–2 (lanes 1–2) were collected and were taken as the lipid raft fractions.

### 2.7 Immunofluorescence microscopy

BGC823 and MGC803 cells were seeded and treated in Lab‐Tek chamber slides and then fixed in 3.3% paraformaldehyde for 25 min, permeabilized with 0.2% Triton X‐100 for 10 min, and blocked with 5% BSA for 2 h. For staining, the cells were primed with anti‐ubiquitin (linkage‐specific K48) rabbit antibody (Alexa Fluor 568) and anti‐caspase‐8 mouse antibody for 1 h and then incubated overnight at 4 °C. The next day, Alexa Fluor 488 goat anti‐mouse IgG was added and incubated for 2 h at room temperature in the dark. Finally, the cells were mounted using the SlowFade Antifade Kit (Thermo Fisher Scientific, Waltham, MA, USA) and analyzed by confocal fluorescence microscopy (FV1000SSIM/IX81; OLYMPUS, Tokyo, Japan).

### 2.8 RNA and miRNA interference

TRAF2 siRNA, Cbl‐b siRNA, c‐Cbl siRNA, CUL3 siRNA, FLIP/L siRNA, and miR‐141 mimic were obtained from Guangzhou RiboBio Co. Ltd (Guangzhou, China). TRAF2 siRNA was synthesized: 5′‐GGACCAAGACAAGAUUGAATT‐3′ (sense) and 5′‐UUCAAUCUUGUCUUGGUCCTT‐3′ (antisense). Cbl‐b siRNA was synthesized: 5′‐GGAUGUGUUUGGGACUAAUTT‐3′ (sense) and 5′‐AUUAGUCCCAAACACAUCCTT‐3′ (antisense). c‐Cbl siRNA was synthesized: 5′‐GGAGACACAUUUCGGAUUATT‐3′ (sense) and 5′‐UAAUCCGAAAUGUGUCUCCTT‐3′ (antisense). CUL3 siRNA was synthesized: 5′‐CCAGCGUAAGAAUAACAGUTT‐3′ (sense) and 5′‐ACUGUUAUUCUUACGCUGGAT‐3′ (antisense). FLIP/L siRNA was synthesized: 5′‐GAAUCUGCCUGAUAAUCGATT‐3′ (sense) and 5′‐UCGAUUAUCAGGCAGAUUCCT‐3′ (antisense). In addition, we found that TRAF2 position 31 glycyl lysine isopeptide was responsible for ubiquitination by Uniport website; a TRAF2 deletion mutation plasmid [TRAF2 Tu31 mutant‐type (MT)] and a TRAF2 wild‐type plasmid (TRAF2 WT) were provided by Obio Technology Corp Ltd (Shanghai, China). To clarify whether DR5 position 339–422 (death domain, DD) was responsible for Cbl‐b or c‐Cbl binding to DR5, a DR5 deletion mutation plasmid (DR5 DD MT) and a DR5 wild‐type plasmid (DR5 WT) were also provided by Obio Technology Corp Ltd. RNA and miRNA interferences were performed as previously described (Xu *et al*., 2009; Zhang *et al*., 2015a).

### 2.9 *In situ* proximity ligation assay

Duolink *in situ* PLA (Olink Bioscience, Uppsala, Sweden) was used to detect the interactions between DR5, Cbl‐b, c‐Cbl, TRAF2, and caspase‐8. Immunofluorescence was performed as previously described (Xu *et al*., 2014). In the assay, oligonucleotide‐conjugated ‘PLA probe’ antibodies are directed against primary antibodies for DR5, Cbl‐b, c‐Cbl, TRAF2, and caspase‐8. Annealing of the ‘PLA probes’ occurs when DR5, Cbl‐b, c‐Cbl, TRAF2, and caspase‐8 are in close proximity, which initiates the amplification of repeat sequences recognized by the fluorescently labeled oligonucleotide probe. For detection, Duolink detection kit 563 was used. The specimens were observed using a confocal fluorescence microscopy (FV1000S‐SIM/IX81, Japan).

### 2.10 RT‐PCR

Total RNA was extracted using RNeasy mini kit as described by the manufacturer, as previously described by Qu *et al*. (2009). The forward primer for Cbl‐b was 5′‐CGCTTGACATCACTGAAGGA‐3′. The reverse primer for Cbl‐b was 5′‐CTTGCCACACTCTGTGCATT‐3′. The forward primer for c‐Cbl was 5′‐TGCCAAAACTGCCACCTGGGG‐3′. The reverse primer for c‐Cbl was 5′‐GGGCTGCGGCCAAATTCCCT‐3′. The forward primer for GAPDH was 5′‐GTGGGGCGCCCCAGGCACCA‐3′. The reverse primer for GAPDH was 5′‐CTCCTTAATGTCACGCACGATTTC‐3′. For Cbl‐b, PCR conditions were 95 °C for 5 min; 30 cycles of 95 °C for 30 s, 59 °C for 30 s, and 72 °C for 30 s; one cycle at 72 °C for 10 min. For c‐Cbl and GAPDH, PCR conditions were 95 °C for 5 min; 33 cycles of 95 °C for 30 s, 56 °C for 45 s, 72 °C for 45 s; one cycle of 72 °C for 10 min. The amplified products were then separated on 1.5% agarose gels, stained with ethidium bromide, and visualized under UV illumination.

### 2.11 qRT‐PCR

As previously described by Li *et al*. (2014), relative expression of microRNA was calculated via the comparative cycle threshold method, and the expression of U6 small nuclear RNA was used as a reference. The forward primer for miR‐141 was 5′‐CGGTAACACTGTCTGGTAAAGATGG‐3′. The PCR conditions were 10 min at 95 °C, followed by 45 cycles at 95 °C for 15 s and 58 °C for 34 s.

### 2.12 Luciferase reporter gene assays

The cells were transfected with miR‐141 mimic and the reporter constructs containing mutant or intact sequence of Cbl‐b or c‐Cbl. Luciferase activity was assayed after transfection for 72 h by the dual luciferase reporter assay system (Promega, Fitchburg, WI, USA) according to the manufacturer's protocol.

### 2.13 Statistical analysis

Data were analyzed in three independent experiments, and the presented data were shown as means ± SD. spss 18.0 computer software (IBM, Armonk, NY, USA) was used for statistical analysis, and *P *<* *0.05 was considered to be a statistically significant result.

## 3 Results

### 3.1 TRAIL induces K48‐linked polyubiquitination and degradation of caspase‐8 in TRAIL‐resistant gastric cancer cells

**Figure 1:**
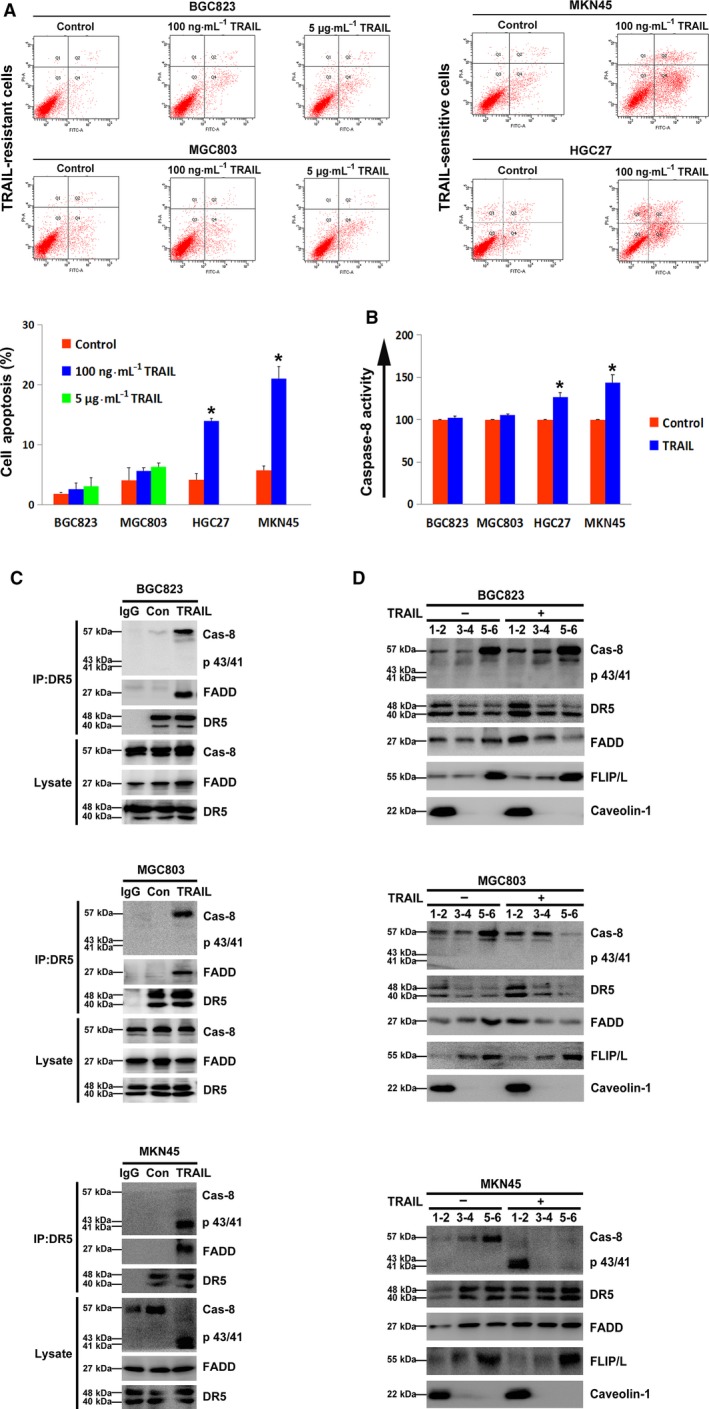
TRAIL did not induce DISC formation in TRAIL‐resistant gastric cancer cells. (A) BGC823 and MGC803 cells were incubated with 100 ng·mL^−1^ or 5 μg·mL^−1^ TRAIL for 24 h. MKN45 and HGC27 cells were incubated with 100 ng·mL^−1^ TRAIL for 24 h. Cell apoptosis was quantified by flow cytometry. *Incubated with TRAIL vs that without TRAIL,* P *<* *0.05. (B) BGC823, MGC803, MKN45, and HGC27 cells were incubated with 100 ng·mL^−1^ TRAIL for 24 h. Caspase‐8 activities were assayed by absorption spectroscopy and expressed relative to the control, designated as 100. *Incubated with TRAIL vs that without TRAIL,* P *<* *0.05. (C) BGC823, MGC803, and MKN45 cells were incubated with 100 ng·mL^−1^ TRAIL for 16 h. The interactions between different proteins were detected by western blot and immunoprecipitation. IgG was used as negative control. (D) BGC823, MGC803, and MKN45 cells were treated with 100 ng·mL^−1^ TRAIL for 30 min, then lysed, and fractionated by the ultracentrifugation. Locations of lipid rafts (lanes 1–2) were determined using caveolin‐1. The indicated proteins were analyzed by western blot.

To examine the mechanism underlying TRAIL resistance in gastric cancer cells, we used BGC823 and MGC803 gastric cancer cells, which we previously demonstrated were not sensitive to TRAIL (Xu *et al*., 2013a,b). We confirmed that either 100 ng·mL^−1^ or 5 μg·mL^−1^ TRAIL did not induce obvious apoptosis in BGC823 and MGC803 cells. In comparison, 100 ng·mL^−1^ TRAIL induced significant apoptosis in the MKN45 and HGC27 gastric cell lines (Fig. 1A). In addition, BGC823 and MGC803 cells showed no changes in caspase‐8 activity in response to TRAIL, while the enzyme activity of caspase‐8 was also found to be increased in MKN45 and HGC27 cells (Fig. 1B).

In untreated TRAIL‐resistant BGC823 and MGC803 cells, we did not detect any interaction among components of the DISC complex, with no interaction between DR5 and FADD or caspase‐8 detected (Fig. 1C). However, TRAIL treatment induced the interactions between DR5 and FADD as well as caspase‐8. Notably, the p43/41 products of caspase‐8 were not detected in BGC823 and MGC803 cells but were observed in MKN45 cells, indicating the initiation of apoptosis (Fig. 1C). Lipid raft extraction experiments in lysates also showed the translocation of DR5, FADD, and caspase‐8 into the lipid raft fractions induced by TRAIL (lanes 1–2, Fig. 1D). And the content of DR5, FADD, and caspase‐8 in the nonlipid raft fractions (lanes 5–6, Fig. 1D) and the middle fractions (lanes 3–4, Fig. 1D) was found to be decreased after TRAIL treatment. The FLIP/L is an endogenous inhibitor of caspase‐8 that negatively interferes with DISC formation (Yu *et al*., 2009). In the present study, the cleavage of caspase‐8 and the translocation of FLIP/L were not detected in BGC823 and MGC803 cells in response to TRAIL. In comparison, in MKN45 cells, the DISC complex formation, containing DR5, FADD, and cleaved caspase‐8, was detected after TRAIL treatment (Fig. 1C,D). These results indicated that TRAIL resistance in BGC823 and MGC803 cells was associated with the absence of DISC formation and function.

**Figure 2:**
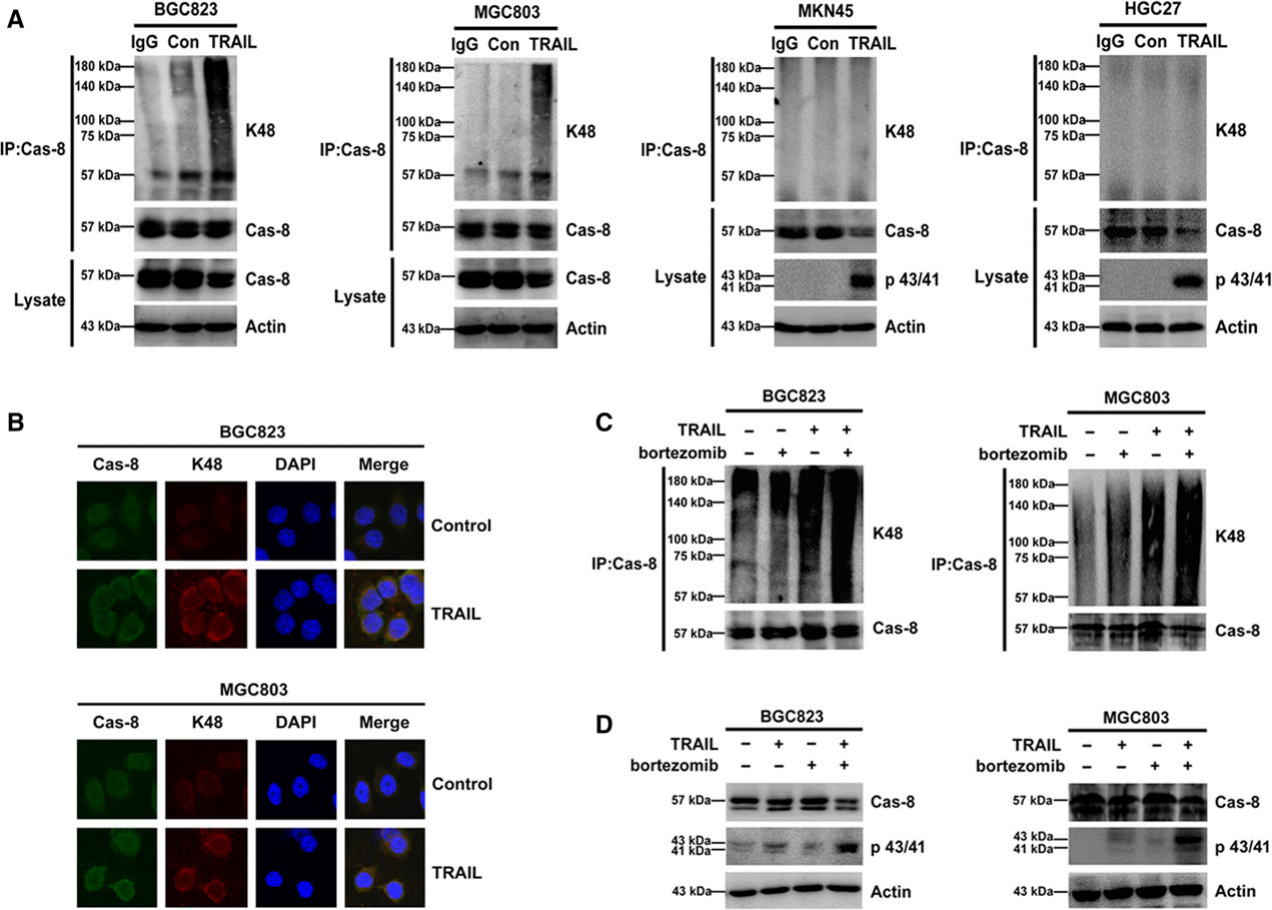
TRAIL induced K48‐linked polyubiquitination and degradation of caspase‐8 in TRAIL‐resistant gastric cancer cells. (A) BGC823, MGC803, MKN45, and HGC27 cells were incubated with 100 ng·mL^−1^ TRAIL for 4 h. The K48‐linked polyubiquitination of caspase‐8 was analyzed by western blot and immunoprecipitation. IgG was used as negative control. (B) BGC823 and MGC803 cells were treated with 100 ng·mL^−1^ TRAIL for 4 h. Then, the cells were stained with anti‐ubiquitin (linkage‐specific K48) rabbit antibody (Alexa Fluor 568) and anti‐caspase‐8 mouse antibody for 1 h, and then incubated overnight at 4 °C. The next day, Alexa Fluor 488 goat anti‐mouse IgG was added and incubated for 1 h at room temperature in the dark. After DAPI nuclear staining, the cells were analyzed by confocal fluorescence microscopy (magnification ×60). (C) BGC823 and MGC803 cells were preincubated with 50 nm bortezomib for 2 h, followed by 100 ng·mL^−1^ TRAIL for 4 h. The K48‐linked polyubiquitination of caspase‐8 was detected by western blot and immunoprecipitation. (D) The p43/41 products of caspase‐8 were analyzed by western blot.

We next examined whether caspase‐8 is polyubiquitinated in gastric cancer cells. In all untreated gastric cancer cells examined, we did not observe any K48‐linked polyubiquitination of caspase‐8 (Fig. 2A). However, TRAIL treatment induced the K48‐linked polyubiquitination of caspase‐8 in TRAIL‐resistant gastric cancer cells, but not in TRAIL‐sensitive MKN45 and HGC27 cells (Fig. 2A). We also confirmed colocalization of K48 and caspase‐8 after TRAIL treatment by confocal fluorescence microscopy, indicating the interaction between K48 and caspase‐8 (Fig. 2B).

Because K48‐linked polyubiquitin chains often promote proteasome‐mediated degradation (Zeng *et al*., 2015; Zhang *et al*., 2016), BGC823 and MGC803 cells were treated with the proteasome inhibitor bortezomib along with TRAIL. As shown in Fig. 2C,D, bortezomib markedly enriched both the K48‐linked polyubiquitination of caspase‐8 and the levels of p43/41 fragments of caspase‐8 induced by TRAIL. These data indicate that TRAIL‐induced K48‐linked polyubiquitination and degradation of caspase‐8 restrain caspase‐8 activation and thus result in TRAIL resistance in TRAIL‐resistant gastric cancer cells.

### 3.2 The depletion or mutation of TRAF2 prevents K48‐linked polyubiquitination of caspase‐8 and increases TRAIL sensitivity in gastric cancer cells

**Figure 3:**
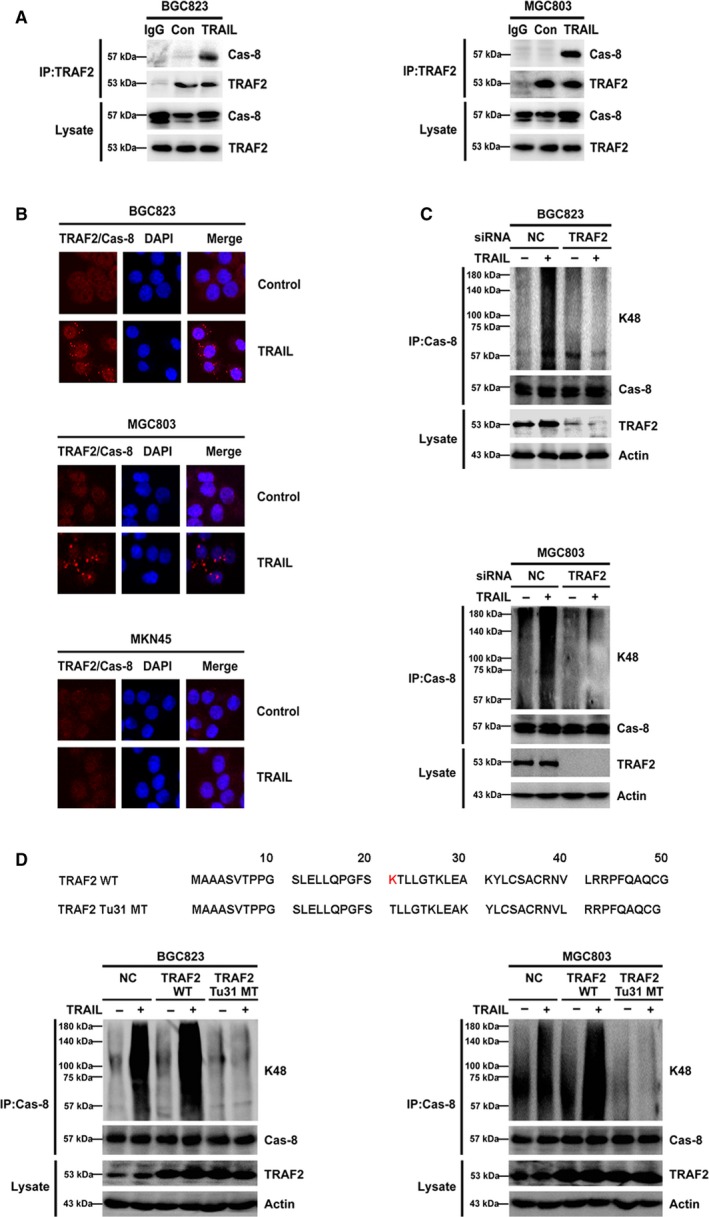
TRAF2 knockdown prevented K48‐linked polyubiquitination of caspase‐8 in TRAIL‐resistant gastric cancer cells. (A) BGC823 and MGC803 cells were incubated with 100 ng·mL^−1^ TRAIL for 4 h. The interaction between TRAF2 and caspase‐8 was detected by western blot and immunoprecipitation. IgG was used as negative control. (B) The interaction between TRAF2 and caspase‐8 in BGC823 cells was also analyzed by Duolink *in situ* PLA. Red, the interaction between TRAF2 and caspase‐8; blue, labeling nucleus using DAPI (magnification ×60). (C) BGC823 and MGC803 cells were transiently transfected with TRAF2 siRNA for 48 h, followed by 100 ng·mL^−1^ TRAIL for 4 h. The K48‐linked polyubiquitination of caspase‐8 was detected by western blot and immunoprecipitation. (D) TRAF2 WT and TRAF2 Tu31 MT sequences were shown (upper). BGC823 and MGC803 cells were transiently transfected with TRAF2 WT or TRAF2 Tu31 MT for 48 h, followed by 100 ng·mL^−1^ TRAIL for 4 h. The K48‐linked polyubiquitination of caspase‐8 was detected by western blot and immunoprecipitation.

As TRAF2 was involved in K48‐linked polyubiquitination of caspase‐8 (Gonzalvez *et al*., 2012), we examined the potential role of TRAF2 in mediating K48‐linked polyubiquitination of caspase‐8 in gastric cancer cells. In untreated cells, we did not detect any interaction between TRAF2 and caspase‐8 (Fig. 3A). However, TRAIL treatment induced the interaction between TRAF2 and caspase‐8 in TRAIL‐resistant BGC823 and MGC803 cells (Fig. 3A). Immunofluorescence also confirmed colocalization of TRAF2 and caspase‐8 in BGC823 and MGC803 cells, but not in TRAIL‐sensitive MKN45 cells (Fig. 3B). To confirm the role of TRAF2 in polyubiquitination of caspase‐8, we silenced TRAF2 in gastric cancer cells and confirmed that TRAF2 siRNA attenuated TRAIL‐induced K48‐linked polyubiquitination of caspase‐8 in TRAIL‐resistant cells (Fig. 3C). TRAF2 position 31 glycyl lysine isopeptide was responsible for caspase‐8 ubiquitination, so we used a TRAF2 deletion mutation plasmid (TRAF2 Tu31 MT). Overexpression of TRAF2 WT increased TRAIL‐induced K48‐linked polyubiquitination of caspase‐8 in gastric cancer cells (Fig. 3D). However, overexpression of TRAF2 Tu31 MT attenuated K48‐linked polyubiquitination of caspase‐8. This indicated that the Tu31 position in TRAF2 was responsible for caspase‐8 ubiquitination.

We next examined whether TRAF2 downstream CUL3 and FLIP/L (Gonzalvez *et al*., 2012; Guiet *et al*., 2002) regulated the K48 ubiquitination of caspase‐8. However, our data showed that the silencing of CUL3 or FLIP/L did not influence K48‐linked polyubiquitination of caspase‐8, and the silencing of CUL3 also did not change the interaction between TRAF2 and caspase‐8 in gastric cancer cells (Fig. S1A,B).

**Figure 4:**
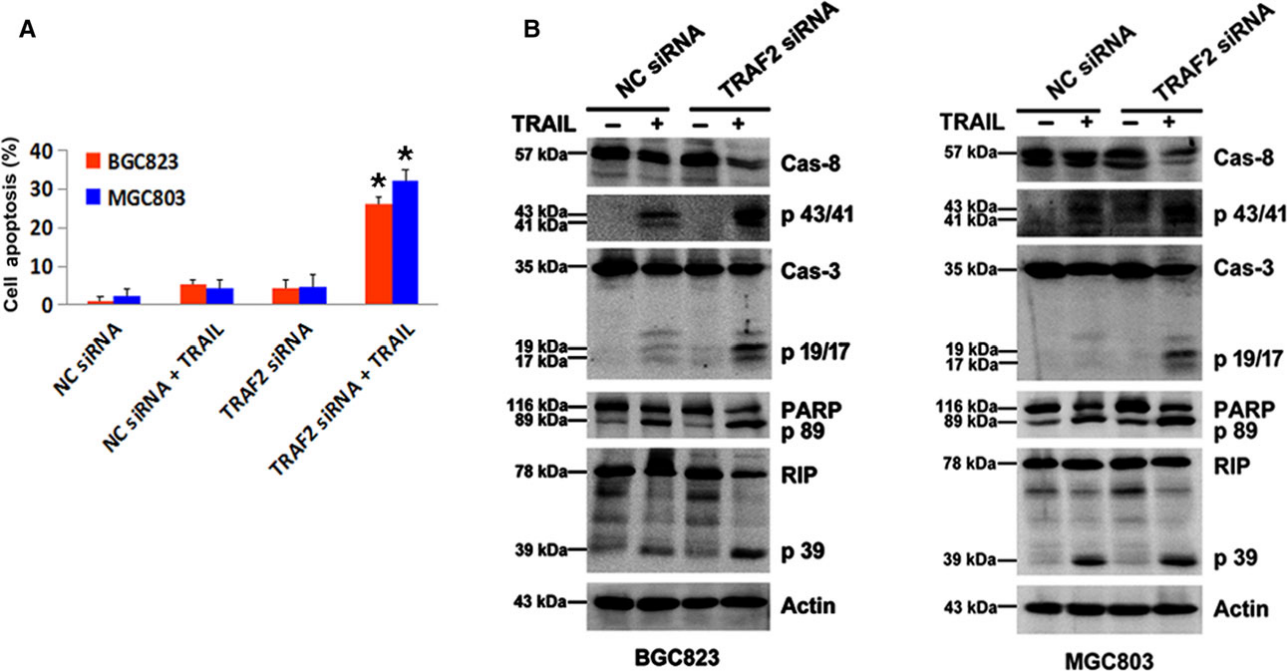
The depletion of TRAF2 expression increased TRAIL sensitivity in gastric cancer cells. (A) BGC823 and MGC803 cells were transiently transfected with TRAF2 siRNA for 48 h, followed by 100 ng·mL^−1^ TRAIL for 24 h. Cell apoptosis was quantified by flow cytometry. *Incubated with TRAIL in TRAF2 siRNA vs that in negative control siRNA (NC siRNA), *P *<* *0.05. (B) The expression of protein was analyzed by western blot.

We also examined the effects of TRAF2 depletion by siRNA on cell apoptosis and found no changes in the apoptotic population in TRAF2 siRNA‐transfected cells (Fig. 4A). However, treatment with TRAF2 siRNA for 48 h followed by TRAIL for 24 h led to increased apoptosis compared with cells transfected with negative control siRNA and treated with TRAIL (Fig. 4A). Furthermore, the p43/41 products of caspase‐8 activated by TRAIL were clearly enhanced in cells transfected with TRAF2 siRNA and treated with TRAIL compared with cells transfected with negative control siRNA and treated with TRAIL (Fig. 4B). The active fragments of caspase 3, PARP, and RIP were also detected. These results suggest that TRAIL promotes the interaction of TRAF2 with caspase‐8 to facilitate the polyubiquitination of caspase‐8, leading to TRAIL resistance in gastric cancer cells. Furthermore, TRAF2 is the ubiquitin ligase responsible for caspase‐8 K48 polyubiquitination.

### 3.3 The depletion of TRAF2 expression did not influence K63‐linked polyubiquitination of caspase‐8 in gastric cancer cells

As K63‐linked polyubiquitination of caspase‐8 influenced the sensitivity of glioblastoma to TRAIL (Bellail *et al*., 2012), we next examined whether TRAIL induced K63‐linked polyubiquitination of caspase‐8 in gastric cancer cells and confirmed K63‐linked polyubiquitination of caspase‐8 in TRAIL‐resistant cells, but not in TRAIL‐sensitive MKN45 cells (Fig. S2A). We examined whether TRAF2 was responsible for K63‐linked polyubiquitination of caspase‐8; however, in cells depleted of TRAF2 by siRNA, K63 polyubiquitination of caspase‐8 was not changed, nor was FLIP/L expression (Fig. S2B). Together, this indicates that an ubiquitin ligase other than TRAF2 is responsible for K63‐linked polyubiquitination of caspase‐8.

### 3.4 DR5, Cbl‐b/c‐Cbl, and TRAF2 form a complex in TRAIL‐resistant gastric cancer cells, and Cbl‐b and c‐Cbl are the critical adaptors linking DR5 and TRAF2

**Figure 5:**
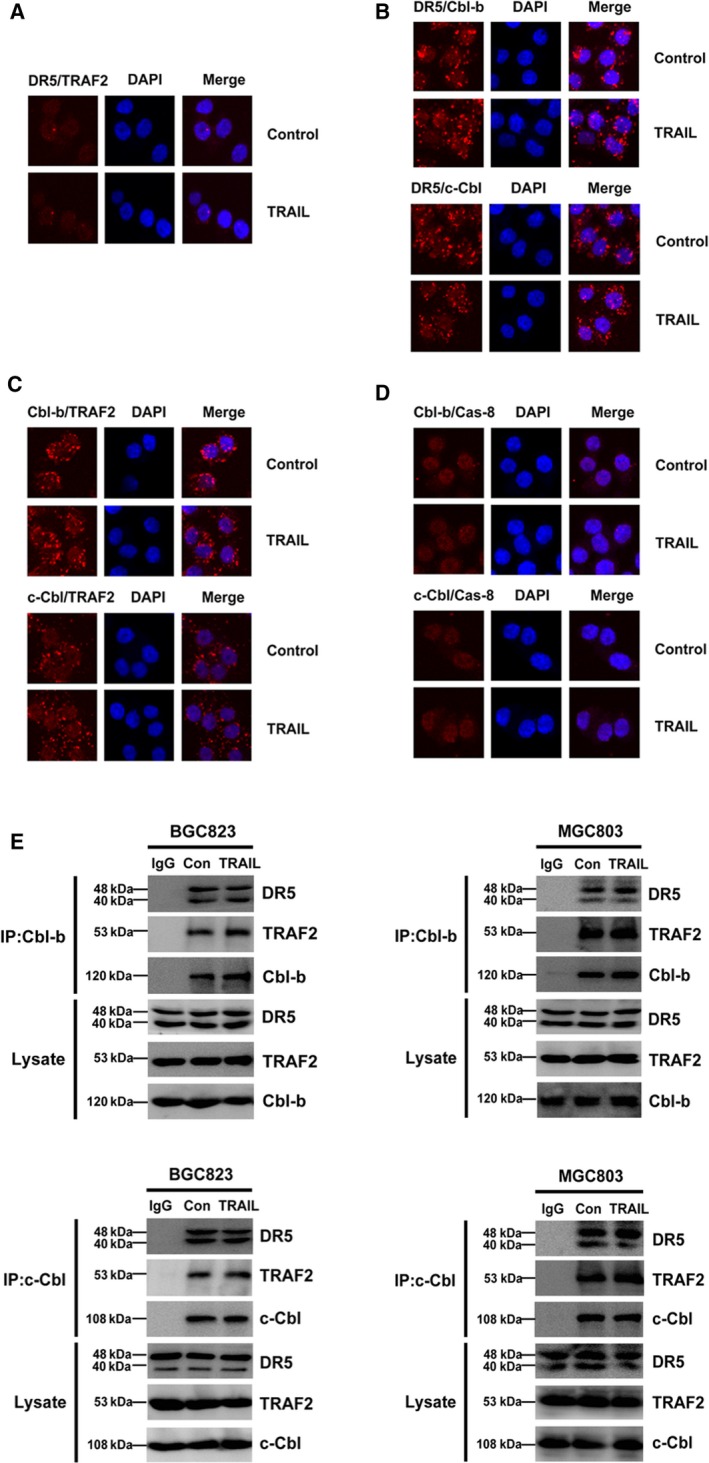
Cbl‐b and c‐Cbl were the critical adaptors linking DR5 and TRAF2 in gastric cancer cells. BGC823 cells were incubated with 100 ng·mL^−1^ TRAIL for 4 h. (A) The interaction between DR5 and TRAF2, (B) the interaction between DR5 and Cbl‐b or c‐Cbl, (C) the interaction between TRAF2 and Cbl‐b or c‐Cbl, (D) the interaction between caspase‐8 and Cbl‐b or c‐Cbl were detected by Duolink *in situ* PLA. Red, the interaction between different proteins; blue, labeling nucleus using DAPI (magnification ×60). (E) BGC823 and MGC803 cells were incubated with 100 ng·mL^−1^ TRAIL for 4 h. The interaction between Cbl‐b and DR5 or TRAF2 and the interaction between c‐Cbl and DR5 or TRAF2 were detected by western blot and immunoprecipitation.

We next performed immunoprecipitation and western blot analysis, but did not observe an interaction between DR5 and TRAF2 in gastric cancer cells without or with TRAIL treatment (Fig. 5A). Our previous study showed that Cbl family proteins regulated TRAIL sensitivity by their translocation into the DISC (Xu *et al*., 2012, 2013a,b). Interestingly, our present data showed that DR5 immunoprecipitated with Cbl‐b and c‐Cbl, and these interactions were not changed by TRAIL (Fig. 5B,E). Moreover, Cbl‐b or c‐Cbl directly bound to TRAF2, and this binding was unaffected by TRAIL (Fig. 5C,E). However, while TRAIL induced the interaction between TRAF2 and caspase‐8 (Fig. 3A,B), it did not promote the interaction between caspase‐8 and Cbl‐b or c‐Cbl (Fig. 5D). Thus, these results suggest that DR5‐Cbl‐b/c‐Cbl‐TRAF2 complex is present in TRAIL‐resistant gastric cancer cells and Cbl‐b and c‐Cbl are the critical adaptors linking DR5 and TRAF2.

To clarify whether DR5 position 339–422 (DD) was responsible for Cbl‐b or c‐Cbl binding to DR5, we used a DR5 deletion mutation plasmid (DR5 DD MT). Overexpression of DR5 WT increased the interaction between DR5 and Cbl‐b or c‐Cbl in gastric cancer cells (Fig. S3). However, overexpression of the DR5 DD MT did not affect binding between DR5 DD MT and Cbl‐b or c‐Cbl, indicating that the DR5 DD region is not responsible for the interaction between DR5 and Cbl‐b or c‐Cbl.

### 3.5 Cbl‐b and c‐Cbl localized in the DR5‐Cbl‐b/c‐Cbl‐TRAF2 complex promote TRAF2‐mediated polyubiquitination of caspase‐8

**Figure 6:**
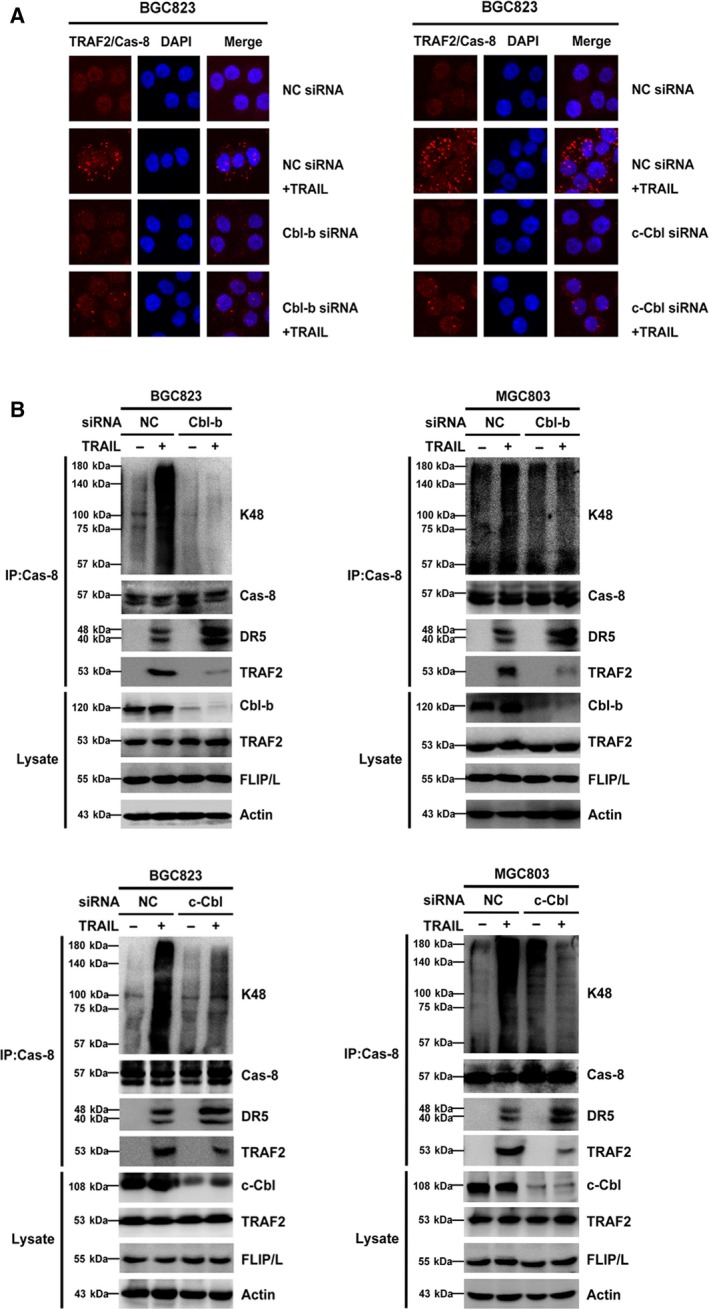
The depletion of Cbl‐b or c‐Cbl expression prevented the interaction between TRAF2 and caspase‐8, and K48‐linked polyubiquitination of caspase‐8 in gastric cancer cells. BGC823 and MGC803 cells were transiently transfected with Cbl‐b or c‐Cbl siRNA for 48 h, followed by 100 ng·mL^−1^ TRAIL for 4 h. (A) The interaction between TRAF2 and caspase‐8 in BGC823 cells was detected by Duolink *in situ* PLA. Red, the interaction between different proteins; blue, labeling nucleus using DAPI (magnification ×60). (B) The K48‐linked polyubiquitination of caspase‐8 was detected by western blot and immunoprecipitation. The expression of protein was analyzed by western blot.

To understand the detailed mechanism of Cbl‐b and c‐Cbl influencing TRAIL sensitivity in gastric cancer cells, we used plasmids with siRNA targeting Cbl‐b or c‐Cbl to knockdown Cbl‐b and c‐Cbl in gastric cancer cells. Although knockdown of Cbl‐b or c‐Cbl did not change the expression of TRAF2 or FLIP/L in TRAIL‐treated cells (Fig. 6B), Cbl‐b or c‐Cbl knockdown did decrease the TRAIL‐mediated interaction between caspase‐8 and TRAF2 and the polyubiquitination of caspase‐8 and promoted the interaction between caspase‐8 and DR5 (Fig. 6A,B).

**Figure 7:**
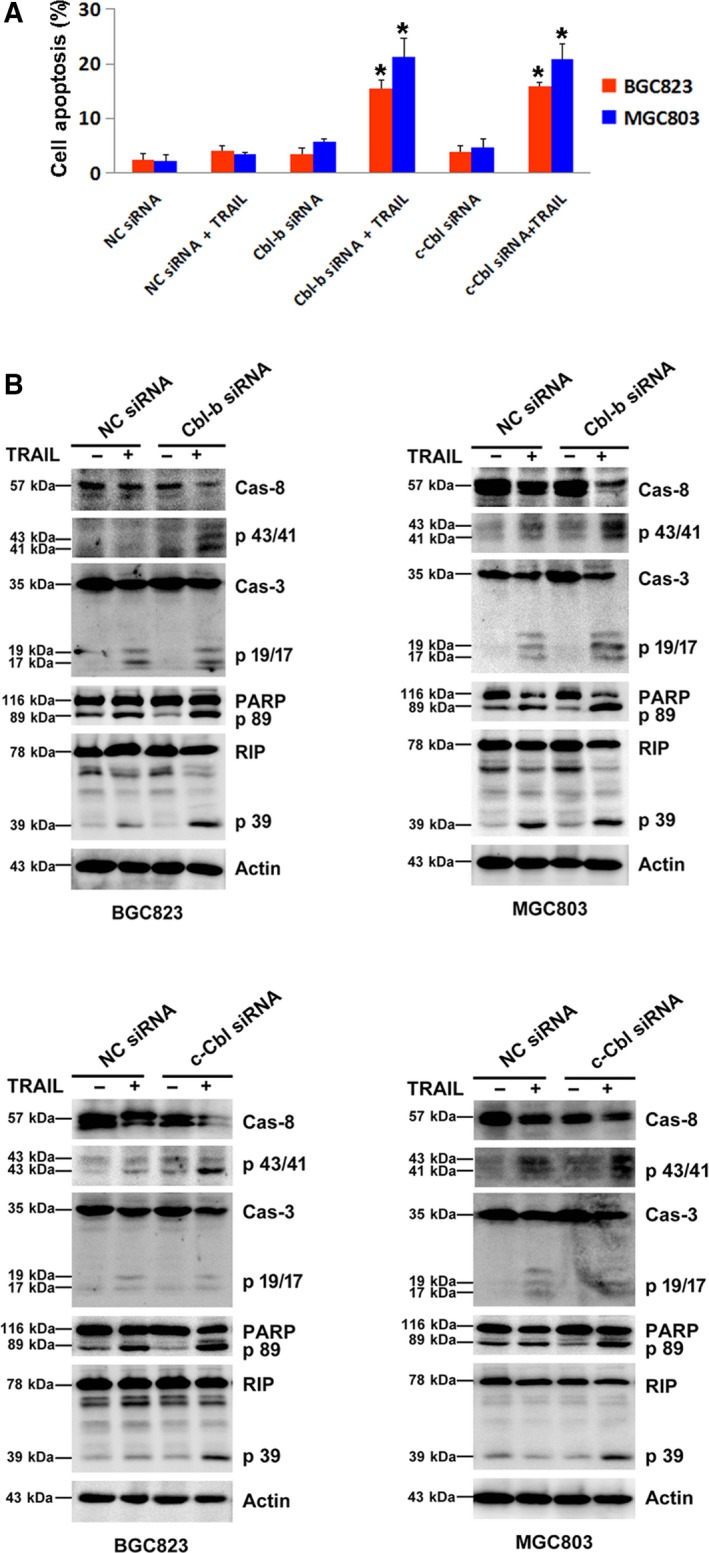
The depletion of Cbl‐b or c‐Cbl expression increased TRAIL sensitivity in gastric cancer cells. BGC823 and MGC803 cells were transiently transfected with Cbl‐b or c‐Cbl siRNA for 48 h, followed by 100 ng·mL^−1^ TRAIL for 24 h. (A) Cell apoptosis was quantified by flow cytometry. *Incubated with TRAIL in Cbl‐b or c‐Cbl siRNA vs that in negative control siRNA (NC siRNA), *P *<* *0.05. (B) The expression of proteins was analyzed by western blot.

In Cbl‐b‐ or c‐Cbl‐knockdown cells, TRAIL promoted increased apoptosis compared with controls (Fig. 7A). The p43/41 products of caspase‐8 from TRAIL activation were enhanced in Cbl‐b‐ or c‐Cbl‐knockdown cells compared with controls, and the active fragments of caspase‐3, PARP, and RIP were detected (Fig. 7B). Thus, these data indicate that Cbl‐b and c‐Cbl localized in the DR5‐Cbl‐b/c‐Cbl‐TRAF2 complex promote TRAF2‐mediated polyubiquitination of caspase‐8, which leads to TRAIL resistance in gastric cancer cells.

### 3.6 miR‐141 by targeting Cbl‐b and c‐Cbl prevents TRAF2‐mediated polyubiquitination of caspase‐8 and enhances TRAIL sensitivity in gastric cancer cells

**Figure 8:**
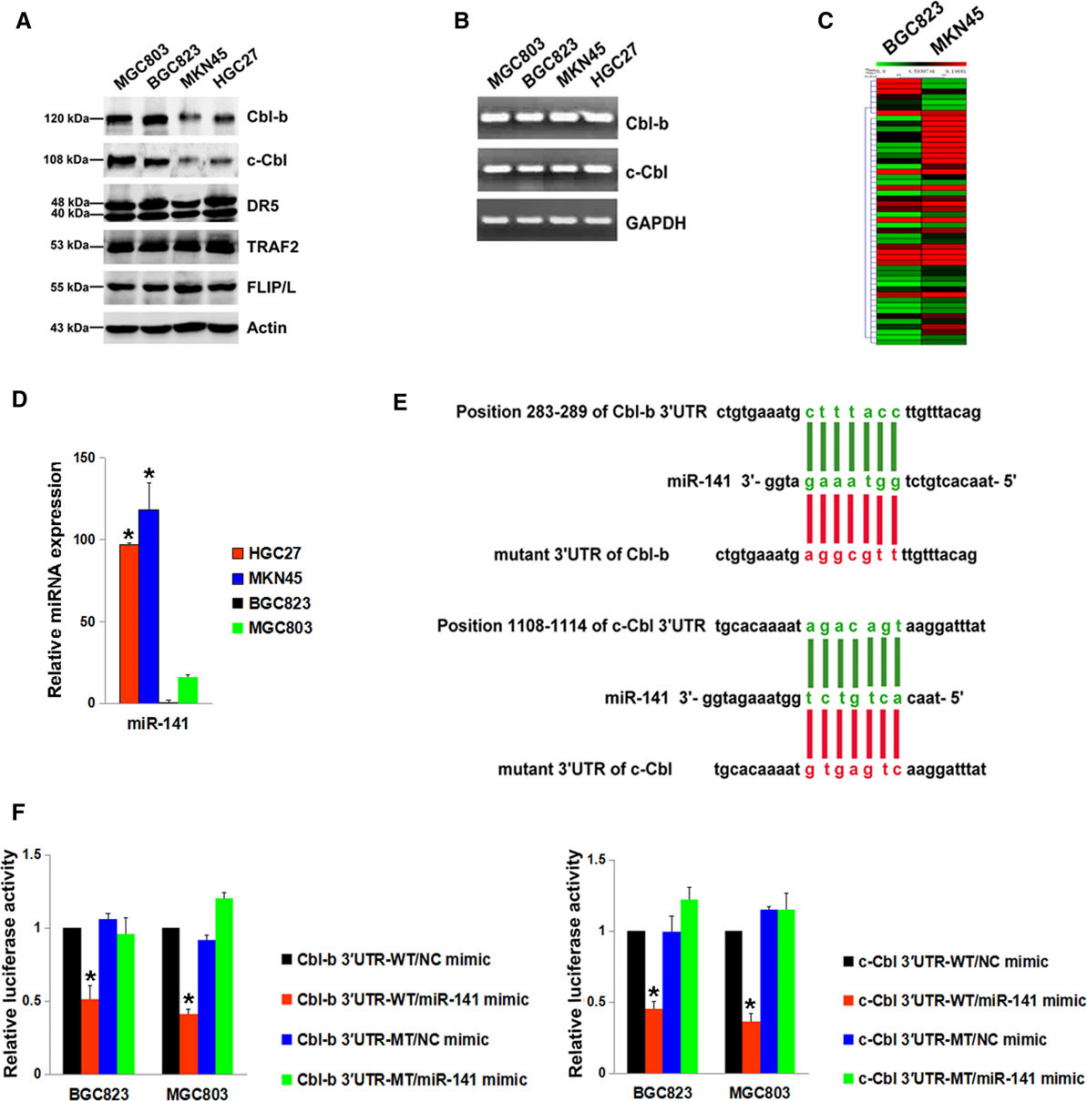
Cbl‐b and c‐Cbl were the targets of miR‐141 in gastric cancer cells. (A) The expression of proteins in TRAIL‐resistant and TRAIL‐sensitive gastric cancer cells was analyzed by western blot. (B) The mRNA abundance of Cbl‐b and c‐Cbl was analyzed by RT‐PCR. (C) Affymetrix miRNA chip showed the differential expression of miRNA in BGC823 and MKN45 cells. (D) The differential expression of miR‐141 was analyzed by qRT‐PCR. *miR‐141 expression in HGC27 or MKN45 cells vs that in BGC823 and MGC803 cells, *P *<* *0.05. (E) Potential binding pattern of miR‐141 to the 3′ UTR of Cbl‐b or c‐Cbl, respectively, and the construct information of mutant 3′ UTR of Cbl‐b or c‐Cbl. (F) Luciferase reporters assay through cotransfecting Cbl‐b or c‐Cbl WT‐Luc, or Cbl‐b or c‐Cbl MT‐Luc with a renilla luciferase control plasmid (pRL‐TK), along with negative control mimic (NC mimic) or miR‐141 mimic. The relative luciferase activities normalized to NC mimic group were shown, *P <* *0.05.

To further identify the effect of DR5‐Cbl‐b/c‐Cbl‐TRAF2 complex on TRAIL sensitivity, we examined the expression of these proteins in TRAIL‐resistant (BGC823 and MGC803) and TRAIL‐sensitive (HGC27 and MKN45) gastric cancer cells (Fig. 8A). Surprisingly, only Cbl‐b and c‐Cbl showed differences in expression between the two cell types and were expressed at low levels in TRAIL‐sensitive cells compared with resistant cells. However, mRNA levels of Cbl‐b and c‐Cbl showed no changes between the two cell types (Fig. 8B). To examine the reasons for the differential expression of Cbl‐b and c‐Cbl, we used Affymetrix miRNA chip to analyze the possible miRNA regulating Cbl‐b and c‐Cbl (Fig. 8C, Table S1). qRT‐PCR revealed elevated levels of miR‐141 in MKN45 and HGC27 cells, suggesting potential support for its regulation of Cbl‐b and c‐Cbl in these cells (Fig. 8D). To evaluate whether Cbl‐b and c‐Cbl were regulated by miR‐141 binding to their 3′ UTR, we constructed luciferase reporters containing either WT or MT miR‐141 binding sites of Cbl‐b and c‐Cbl (Fig. 8E). As shown in Fig. 8F, overexpression of miR‐141 decreased the luciferase activity of the WT reporter, but not the MT reporter. Together, these data suggest that Cbl‐b and c‐Cbl may be targets of miR‐141 in gastric cancer cells.

**Figure 9:**
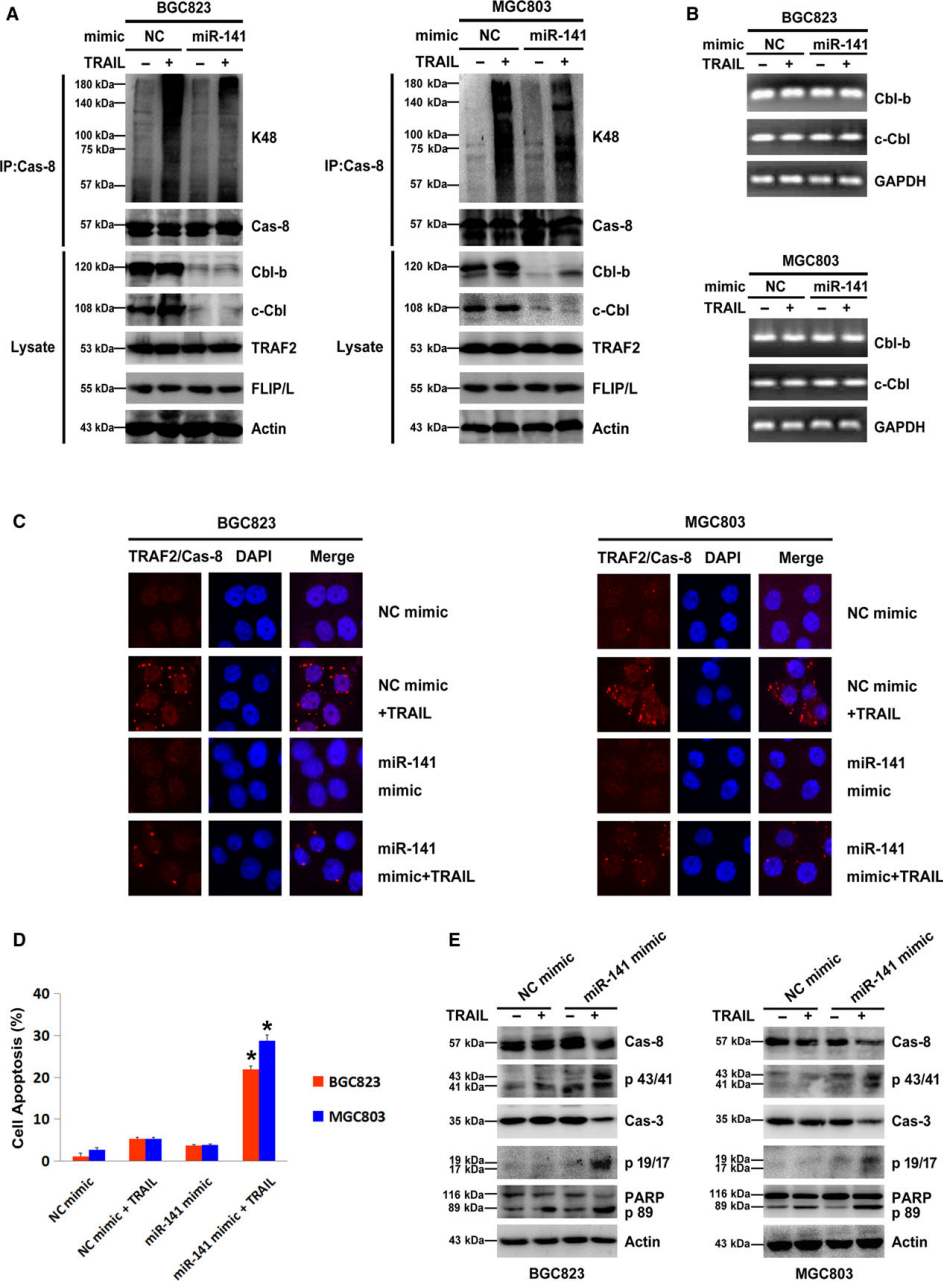
Overexpression of miR‐141 prevented K48‐linked polyubiquitination of caspase‐8 and increased TRAIL sensitivity in gastric cancer cells. (A) BGC823 and MGC803 cells were transiently transfected with miR‐141 mimic for 48 h, followed by 100 ng·mL^−1^ TRAIL for 4 h. The K48‐linked polyubiquitination of caspase‐8 was detected by western blot and immunoprecipitation. The expression of protein was analyzed by western blot. (B) The mRNA abundance of Cbl‐b and c‐Cbl was analyzed by RT‐PCR. (C) The interaction between TRAF2 and caspase‐8 was detected by Duolink *in situ* PLA. Red, the interaction between different proteins; blue, labeling nucleus using DAPI (magnification ×60). (D) BGC823 and MGC803 cells were transiently transfected with miR‐141 mimic for 48 h, followed by 100 ng·mL^−1^ TRAIL for 24 h. Cell apoptosis was quantified by flow cytometry. *Incubated with TRAIL in miR‐141 mimic vs that in negative control mimic (NC mimic), *P *<* *0.05. (E) The expression of proteins was analyzed by western blot.

To determine whether miR‐141 influenced TRAIL sensitivity by targeting Cbl‐b and c‐Cbl, a miR‐141 mimic was transfected into resistant gastric cancer cells (Fig. 9A). Compared with the negative control mimic, overexpression of miR‐141 inhibited the protein expression levels of Cbl‐b and c‐Cbl. However, the mRNA abundance of Cbl‐b and c‐Cbl was not changed by overexpression of miR‐141 mimic (Fig. 9B). Overexpression of miR‐141 did not change the expression of TRAF2 and FLIP/L (Fig. 9A), but importantly it prevented the interaction between TRAF2 and caspase‐8 (Fig. 9C) and the K48‐linked polyubiquitination of caspase‐8 (Fig. 9A). Moreover, the p43/41 products of caspase‐8 activated by TRAIL were enhanced in cells transfected with miR‐141 mimic (Fig. 9E). As shown in Fig. 9D, the miR‐141 mimic had little effect on apoptosis. However, preincubation with the miR‐141 mimic for 48 h followed by TRAIL for 24 h led to increased apoptosis compared to the negative control. Furthermore, the active fragment of caspase‐3 and PARP was also detected (Fig. 9E). The results suggest that miR‐141 targets Cbl‐b and c‐Cbl to prevent TRAF2‐mediated polyubiquitination and degradation of caspase‐8, and thus increases TRAIL sensitivity in gastric cancer cells.

## 4 Discussion

Previous reports showed that DISC formation is important for TRAIL‐triggered apoptosis (Lin *et al*., 2014). In the present study, TRAIL induced effective DISC formation in TRAIL‐sensitive gastric cancer cells. However, in resistant cells, the ineffective DISC did not trigger apoptosis. It has been shown that TRAF2‐mediated K48‐linked polyubiquitination of caspase‐8 prevented TRAIL‐triggered apoptosis in colon cancer cells (Gonzalvez *et al*., 2012). In the present study, TRAIL induced the interaction between TRAF2 and caspase‐8, and then, TRAF2 mediated K48‐linked polyubiquitination of caspase‐8. We also found that the Tu31 position in TRAF2 was responsible for caspase‐8 ubiquitination. The proteasome inhibitor bortezomib markedly enriched this polyubiquitination and the p43/41 products of caspase‐8, indicating that they were degraded by the proteasome. In addition, silencing of TRAF2 attenuated K48‐linked polyubiquitination of caspase‐8 and increased caspase‐8 activation and TRAIL‐induced apoptosis. This indicated that TRAF2 is the critical factor in regulating TRAIL sensitivity in gastric cancer cells.

Although Cbl‐b and c‐Cbl are also E3 ubiquitin ligase, but our data showed that TRAIL did not promote the interaction between caspase‐8 and Cbl‐b or c‐Cbl. Thus, Cbl‐b and c‐Cbl are not responsible for K48‐linked polyubiquitination of caspase‐8. Moreover, in the present study, the silencing of TRAF2 downstream CUL3 or FLIP/L did not influence K48‐linked polyubiquitination of caspase‐8 and the inhibition of CUL3 also did not affect the interaction between TRAF2 and caspase‐8. These results suggest that CUL3 and FLIP/L are not involved in TRAF2‐mediated K48 polyubiquitination of caspase‐8 in gastric cancer cells. In addition, K63‐linked polyubiquitination of caspase‐8 was also detected in TRAIL‐resistant cells, but not in TRAIL‐sensitive MKN45 cells, indicating that K63 polyubiquitination of caspase‐8 may be another resistant factor. However, TRAF2 was not responsible for K63 polyubiquitination of caspase‐8. Above all, TRAF2 was the responsible ubiquitin ligase for the K48 polyubiquitination of caspase‐8, and mediated TRAIL resistance in gastric cancer cells.

What molecules can influence TRAF2‐mediated K48‐linked polyubiquitination of caspase‐8? Previous work has shown that TRAF2 binds to DR5 and is localized to the PLAC in glioblastoma (Bellail *et al*., 2012). However, in the present study, no interaction between DR5 and TRAF2 was detected in gastric cancer cells regardless of TRAIL treatment. Our previous study showed that TRAIL promoted the translocation of Cbl family proteins into the DISC and prevented TRAIL‐triggered apoptosis (Xu *et al*., 2012, 2013a,b). However, whether Cbl‐b and c‐Cbl were vital factors in regulating TRAF2‐mediated polyubiquitination of caspase‐8 was unknown. Our present data showed that although no interaction between DR5 and TRAF2 was detected, DR5 directly bound to Cbl‐b and c‐Cbl, and Cbl‐b and c‐Cbl were the critical adaptors linking DR5 and TRAF2, forming the DR5‐Cbl‐b/c‐Cbl‐TRAF2 complex in gastric cancer cells. Thus, Cbl‐b and c‐Cbl play a function as adaptor protein in the present study. However, we found that the DD position in DR5 was not responsible for the interaction between DR5 and Cbl‐b or c‐Cbl. So, the specific binding sites of DR5 and Cbl‐b or c‐Cbl need to be further explored in the future.

In addition, TRAIL clearly induced the interaction of FADD and caspase‐8 with DR5, but caspase‐8 activation was not detected in the DISC, indicating that the DISC complex was not functioning in TRAIL‐resistant cells. Conversely, TRAIL promoted caspase‐8 translocation into the DR5‐Cbl‐b/c‐Cbl‐TRAF2 complex to interact with TRAF2, and TRAF2 mediated the K48‐linked polyubiquitination of caspase‐8, forming the DISC complex. Moreover, knockdown of Cbl‐b or c‐Cbl decreased the interaction between TRAF2 and caspase‐8 and reduced the polyubiquitination of caspase‐8 to enhance the activation of caspase‐8, restoring TRAIL sensitivity. Although FLIP/L stability and ubiquitination is controlled by c‐Cbl (Zhao *et al*., 2013), and Cbl‐b associates with TRAF2 upon CD40 ligation (Qiao *et al*., 2007), in our study, knockdown of Cbl‐b or c‐Cbl did not change the expression of FLIP/L and TRAF2. In our previous study, we observed that oxaliplatin promoted DISC formation by downregulation of Cbl‐b and c‐Cbl and thus enhanced TRAIL‐induced apoptosis (Xu *et al*., 2009). Our present study further demonstrated that Cbl‐b and c‐Cbl bound to TRAF2 and promoted TRAF2‐mediated polyubiquitination and degradation of caspase‐8, resulting in the inability of DISC to induce apoptosis. This novel finding provides new insights that may help in the identification of the roles of Cbl‐b and c‐Cbl in TRAIL resistance in gastric cancer.

Another important question is why DR5‐Cbl‐b/c‐Cbl‐TRAF2 complex formed in TRAIL‐resistant gastric cancer cells. Our present data showed that in TRAIL‐sensitive cells, Cbl‐b and c‐Cbl, the critical adaptors linking DR5 and TRAF2, were expressed at a low level. Previous studies reported that the hepatitis B virus X protein enhanced the sensitivity of hepatocytes to TRAIL by upregulation of miR‐125a targeting TNFAIP3 to enhance the activation of caspase‐8 (Zhang *et al*., 2015b). Because our present data showed no changes in Cbl‐b and c‐Cbl mRNA in TRAIL‐sensitive and TRAIL‐resistant gastric cancer cells, we performed Affymetrix miRNA chip analyses to identify potential miRNA. qRT‐PCR showed that miR‐141, which likely targets Cbl‐b and c‐Cbl, was highly expressed in TRAIL‐sensitive cells. Our luciferase reporter gene assays showed that Cbl‐b and c‐Cbl were the targets of miR‐141 in gastric cancer cells. Among top five elevated miRNA, miRNA chip analyses indicated that miR‐429 and miR‐200a‐3p were also probable miRNA targeting Cbl family protein. However, further luciferase reporter gene assays showed that Cbl‐b and c‐Cbl were not the targets of miR‐429 and miR‐200a‐3p in gastric cancer cells (data not shown). In addition, although overexpression of miR‐141 did not change the mRNA abundance of Cbl‐b and c‐Cbl, overexpression of miR‐141 inhibited the protein expression of Cbl‐b and c‐Cbl and subsequently prevented the interaction between TRAF2 and caspase‐8 to reduce the polyubiquitination of caspase‐8, which finally increased TRAIL sensitivity.

**Figure 10:**
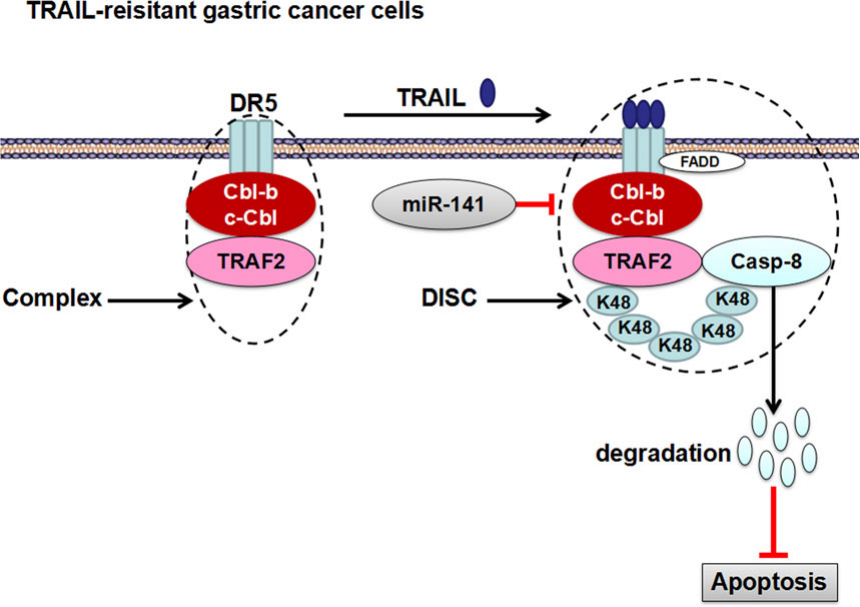
Schematic representation of the proposed model. DR5, Cbl‐b/c‐Cbl, and TRAF2 form a complex in TRAIL‐resistant gastric cancer cells, and Cbl‐b and c‐Cbl are the critical adaptors linking DR5 and TRAF2. TRAIL induces FADD and caspase‐8 translocation into the DR5‐Cbl‐b/c‐Cbl‐TRAF2 complex, and the interaction between caspase‐8 and TRAF2, forming the DISC. TRAF2 then mediates the K48‐linked polyubiquitination and degradation of caspase‐8, which blocks the DISC from inducing apoptosis. miR‐141, by targeting Cbl‐b and c‐Cbl, inhibits TRAF2‐mediated K48‐linked polyubiquitination and degradation of caspase‐8, which leads to the induction of apoptosis and hence increases the sensitivity of TRAIL in gastric cancer cells.

Taken together, our results showed that DR5, Cbl‐b/c‐Cbl, and TRAF2 form a complex in TRAIL‐resistant gastric cancer cells and that Cbl‐b and c‐Cbl are the critical adaptors linking DR5 and TRAF2. TRAIL induces FADD and caspase‐8 translocation into the DR5‐Cbl‐b/c‐Cbl‐TRAF2 complex, and the interaction between caspase‐8 and TRAF2, forming the DISC. TRAF2 then mediates the K48‐linked polyubiquitination and degradation of caspase‐8, which blocks the DISC from inducing apoptosis. miR‐141, by targeting Cbl‐b and c‐Cbl, inhibits TRAF2‐mediated K48‐linked polyubiquitination and degradation of caspase‐8, which leads to the induction of apoptosis and hence increases the sensitivity of TRAIL in gastric cancer cells (Fig. 10). Our study provides new insights to better understand the mechanism of TRAIL resistance in gastric cancer cells. In addition, recombinant human TRAIL and monoclonal agonist antibodies targeted DR4 and DR5 have been evaluated in the clinic. Some Phase 1/1b and Phase 2 studies provided clinical benefit in patients with advanced solid tumors (Ba‐Sang *et al*., 2016; Huang and Sheikh, 2007; Tabernero *et al*., 2015). Through investigating the mechanism of gastric cancer cells resistance to TRAIL, we may screen out the dominant population of TRAIL treatment for gastric cancer in the future.

## Data Accessibility

Research data pertaining to this article are located at figshare.com: https://doi.org/10.6084/m9.figshare.5484358

**Table S1.** Affymetrix miRNA chip showed the differential miRNA expression in TRAIL‐resistant BGC823 and TRAIL‐sensitive MKN45 cells.

## Author contributions

YL and XQ designed research; LX and YZ performed the data acquisition; XC, TG, CL, and BL supervised the data and algorithms; RM, YF, RY, and HY performed data analysis and interpretation; YM, KH, DL, XH, and JG carried out the statistical analysis; LX performed manuscript preparation; YL and XQ participated in manuscript editing and review. All authors read and approved the final manuscript.

## Supporting information


**Fig. S1.** The depletion of either CUL3 or FLIP/L expression did not change K48‐linked polyubiquitination of caspase‐8 in gastric cancer cells.

**Fig. S2.** The depletion of TRAF2 expression did not influence K63‐linked polyubiquitination of caspase‐8 in gastric cancer cells.

**Fig. S3.** DR5 WT and DR5 DD MT sequences were shown (upper).
