# Supplementary figures and images for: DR5‐Cbl‐b/c‐Cbl‐TRAF2 complex inhibits TRAIL‐induced apoptosis by promoting TRAF2‐mediated polyubiquitination of caspase‐8 in gastric cancer cells
Source: Mol Oncol. 2017 Oct 27;11(12):1733–51. doi: 10.1002/1878-0261.12140 (PMC5709619; doi:10.1002/1878-0261.12140)

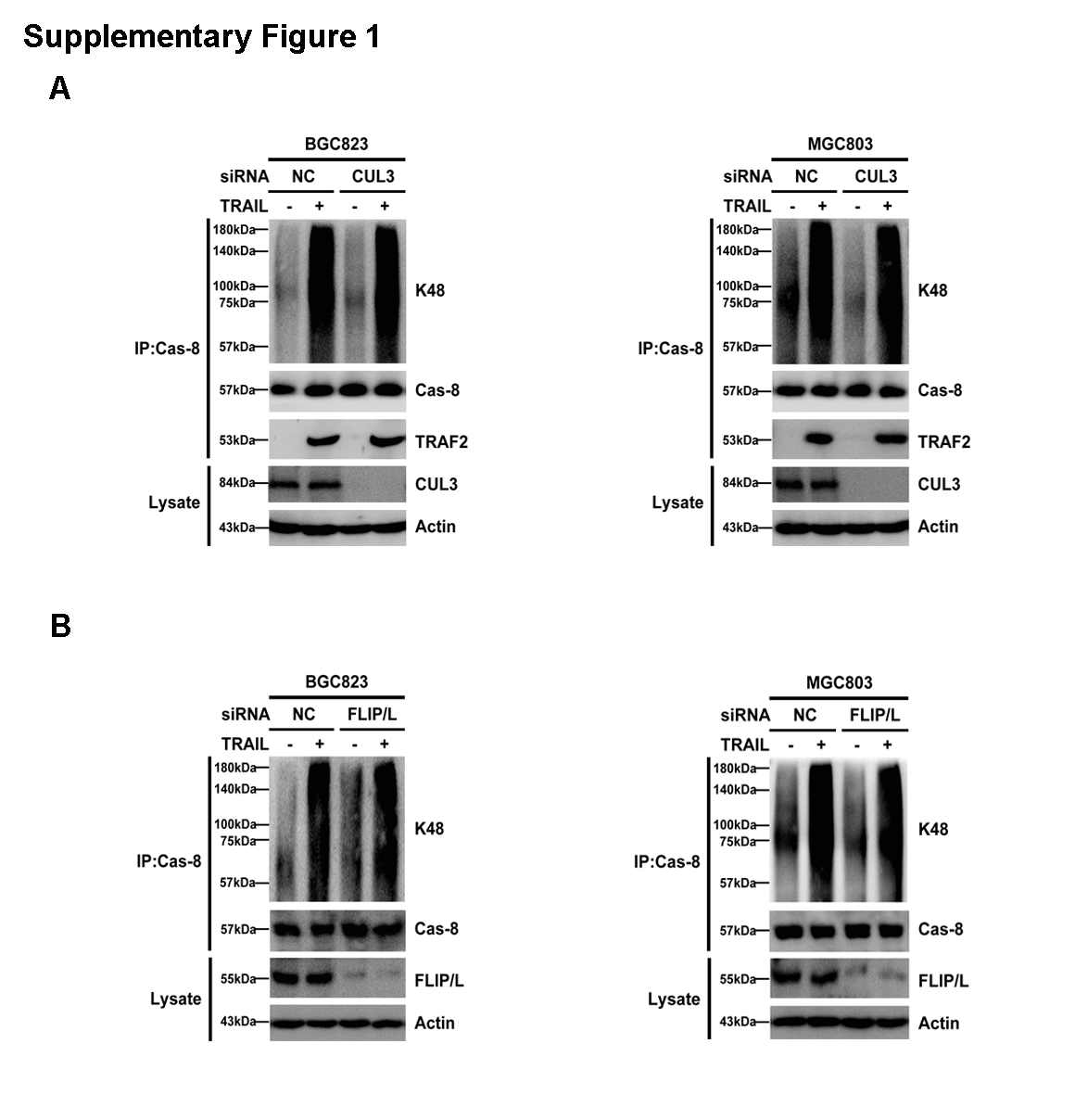

Supplement: Supplementary file 1 — Fig. S1. The depletion of either CUL3 or FLIP/L expression did not change K48‐linked polyubiquitination of caspase‐8 in gastric cancer cells. [file MOL2-11-1733-s001.tif]

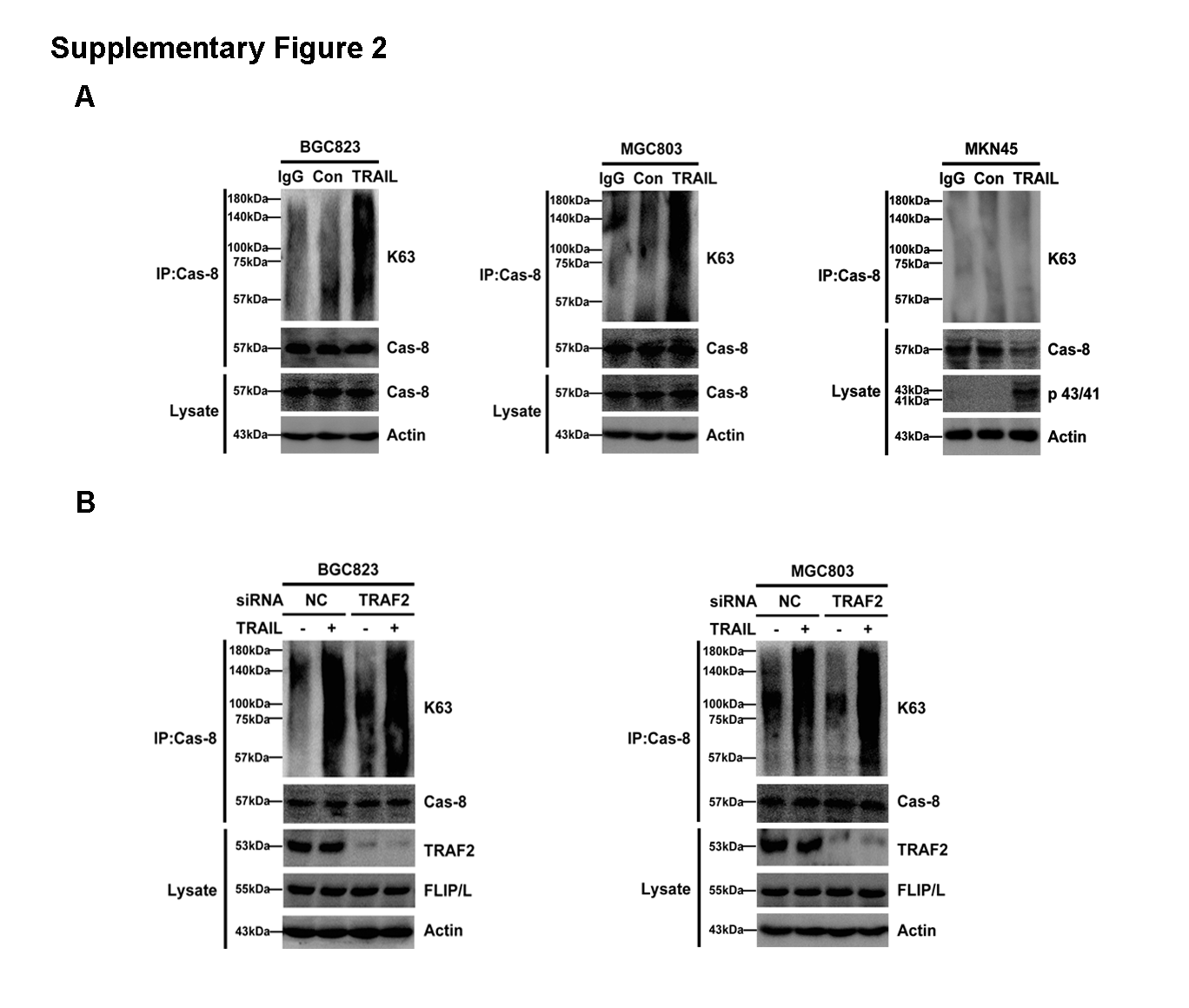

Supplement: Supplementary file 2 — Fig. S2. The depletion of TRAF2 expression did not influence K63‐linked polyubiquitination of caspase‐8 in gastric cancer cells. [file MOL2-11-1733-s002.tif]

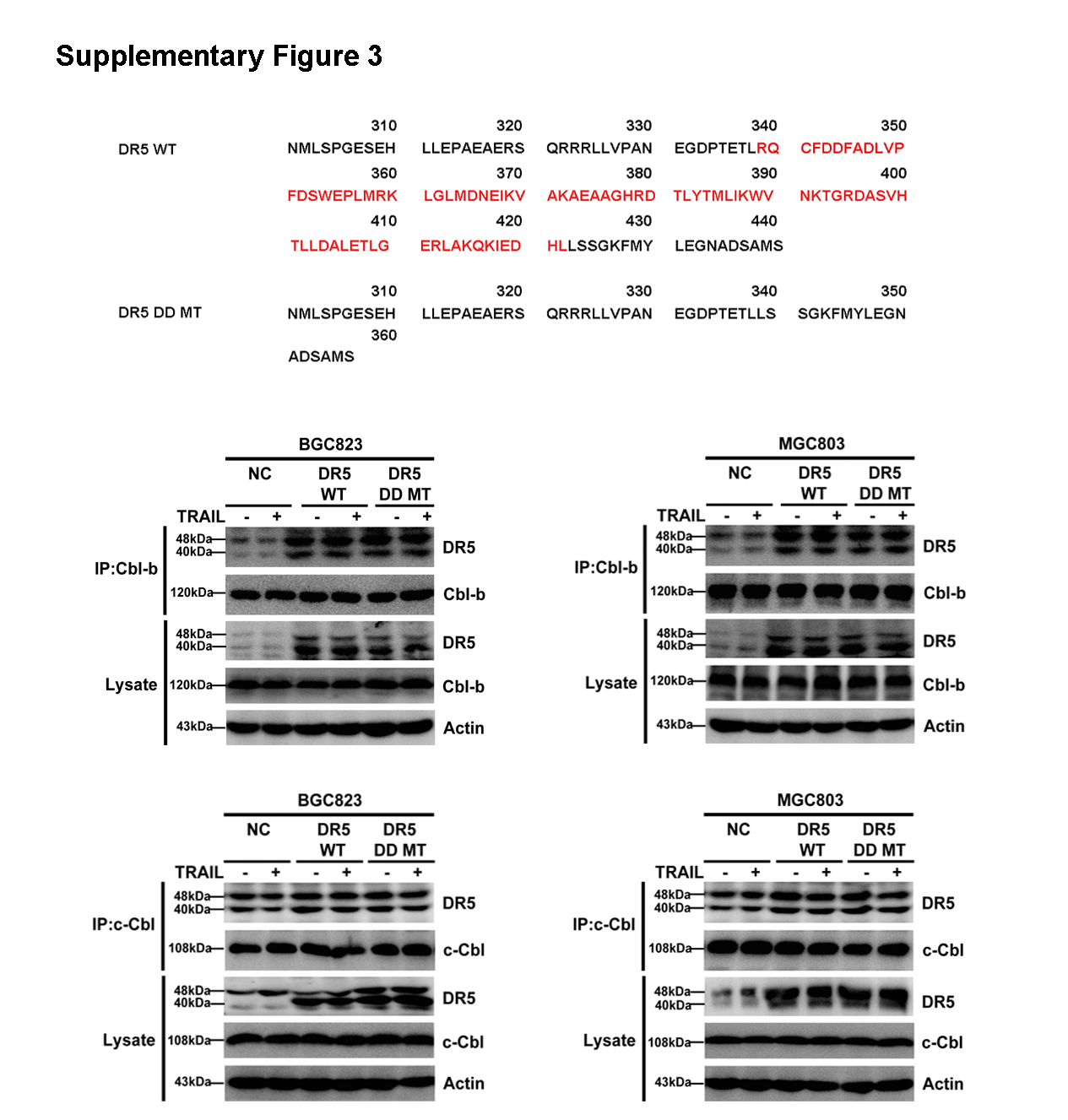

Supplement: Supplementary file 3 — Fig. S3. DR5 WT and DR5 DD MT sequences were shown (upper). [file MOL2-11-1733-s003.tif]
